# In vitro generation of Sertoli-like and haploid spermatid-like cells from human umbilical cord perivascular cells

**DOI:** 10.1186/s13287-017-0491-8

**Published:** 2017-02-15

**Authors:** Ekaterina Shlush, Leila Maghen, Sonja Swanson, Shlomit Kenigsberg, Sergey Moskovtsev, Tanya Barretto, Andrée Gauthier-Fisher, Clifford L. Librach

**Affiliations:** 1 CReATe Fertility Centre, 790 Bay Street, Toronto, Ontario M5N 1G8 Canada; 2 Department of Obstetrics & Gynaecology, University of Toronto, Toronto, Ontario Canada; 3 Department of Physiology, University of Toronto, Toronto, Ontario Canada; 4 Institute of Medical Sciences, University of Toronto, Toronto, Ontario Canada; 5 Department of Gynecology, Women’s College Hospital, Toronto, Ontario Canada

**Keywords:** Ex-vivo spermatogenesis, Germ cell, Sertoli cell, Spermatogonial stem cell, Niche, Human mesenchymal stem cell, Perivascular cell, Human umbilical cord, Xenograft, Paracrine

## Abstract

**Background:**

First trimester (FTM) and term human umbilical cord-derived perivascular cells (HUCPVCs), which are rich sources of mesenchymal stem cells (MSCs), can give rise to Sertoli cell (SC)-like as well as haploid germ cell (GC)-like cells in vitro using culture conditions that recapitulate the testicular niche.

Gamete-like cells have been produced ex vivo using pluripotent stem cells as well as MSCs. However, the production of functional gametes from human stem cells has yet to be achieved.

**Methods:**

Three independent lines of FTM and term HUCPVCs were cultured using a novel 5-week step-wise in vitro differentiation protocol recapitulating key physiological signals involved in testicular development. SC- and GC-associated phenotypical properties were assessed by real-time polymerase chain reaction (RT-PCR), quantitative PCR immunocytochemistry, flow cytometry, and fluorescence in-situ hybridization (FISH). Functional spermatogonial stem cell-like properties were assessed using a xenotranplantation assay.

**Results:**

Within 3 weeks of differentiation, two morphologically distinct cell types emerged including large adherent cells and semi-attached round cells. Both early GC-associated markers (VASA, DAZL, GPR125, GFR1α) and SC-associated markers (FSHR, SOX9, AMH) were upregulated, and 5.7 ± 1.2% of these cells engrafted near the inner basal membrane in a xenograft assay. After 5 weeks in culture, 10–30% of the cells were haploid, had adopted a spermatid-like morphology, and expressed PRM1, Acrosin, and ODF2. Undifferentiated HUCPVCs secreted key factors known to regulate spermatogenesis (LIF, GDNF, BMP4, bFGF) and 10–20% of HUCPVCs co-expressed SSEA4, CD9, CD90, and CD49f. We hypothesize that the paracrine properties and cellular heterogeneity of HUCPVCs may explain their dual capacity to differentiate to both SC- and GC-like cells.

**Conclusions:**

HUCPVCs recapitulate elements of the testicular niche including their ability to differentiate into cells with Sertoli-like and haploid spermatid-like properties in vitro. Our study supports the importance of generating a niche-like environment under ex vivo conditions aiming at creating mature GC, and highlights the plasticity of HUCPVCs. This could have future applications for the treatment of some cases of male infertility.

**Electronic supplementary material:**

The online version of this article (doi:10.1186/s13287-017-0491-8) contains supplementary material, which is available to authorized users.

## Background

The production of mature functional gametes from spermatogonial stem cells and pluripotent stem cells ex vivo or using xenografting techniques has been achieved in rodent models [1], large domestic animals [2], and by allogeneic spermatogonia stem cell (SSC) transplantation into nonhuman primates [3]. To date, studies using various human stem cell sources have yielded rudimentary, but not functional, gametes [4]. However, the influx of recent reports on this topic [5–9] have described key milestones towards achieving this goal and suggest that the derivation of gametes from human pluripotent stem cells might be achieved by mimicking the physiological signals known to regulate spermatogenesis [10, 11]. Normal spermatogenesis is highly dependent on precise environmental cues. Cell autonomous factors, niche crosstalk, and hormones are all required for this regulated process [12]. Spermatogenesis starts in utero where the primordial germ cells (PGCs) create a pool of diploid SSCs which will eventually differentiate to mature haploid sperm after puberty. More than 50 years ago, the Canadian team led by Yves Clermont put forward the hierarchical model of spermatogenesis [13]. This process is now well characterized, and markers have been identified for each of the differentiation stages [12, 14, 15]. The concept that the entire testicular niche is required for ex vivo sperm production was a pivotal factor in the first successful derivation of functional sperm from mouse spermatogonial stem cell ex vivo in a three-dimensional system [1]. Sato et al. [1] plated testicular tissue from neonate rodents and created mature functional sperm and healthy offspring.

The principal somatic cell populations of the mammalian testes niche include Sertoli cells (SCs), Leydig, and peritubular myoid cells. SCs are the only somatic cells located within seminiferous tubules and are intimately associated with developing germ cells (GCs). For these reasons, they are considered to be the most important somatic cell population contributing to the SSC niche [12]. The origin of human SCs remains debatable [16]. Studies in rodents provided direct evidence for an epithelial origin [17, 18], but recent human studies suggested that they may also originate from the mesenchyme [19, 20]. Others have suggested a dual origin for SCs, leading to the production of more than one type of SC during development [21].

Our group has previously characterized the stem cell-associated properties of a rich source of mesenchymal stromal cells (MSCs) derived from first trimester (FTM) umbilical cords [22] and we, as well as others, have characterized these properties in term human umbilical cords [23]. Previous reports demonstrated that these cells, termed human umbilical cord-derived perivascular cells (HUCPVCs), have the capacity to differentiate towards both mesenchymal and nonmesenchymal lineages (all three germ layers) in vitro [22, 23]. To further investigate the plasticity of HUCPVCs, here we aimed to differentiate HUCPVCs towards the SC lineage, with the major aim of recreating an in vitro testicular niche. Interestingly, utilizing a novel protocol that we developed for these cells, we were able to sequentially recapitulate key physiological signals involved in testicular development, and this led to the in vitro differentiation of HUCPVCs towards both haploid GC-like cells as well as SC-like cells.

## Methods

### Ethical approval

Independent research ethics board (REB) approval was obtained for the collection of human umbilical cord (REB No. 454-2011, Sunnybrook Research Institute, Toronto, Canada; REB No. 28889, University of Toronto, Toronto, Canada) and human testicular tissue obtained from orchiectomies performed at Mount Sinai Hospital, Toronto, Canada (REB No. 30252, University of Toronto and REB No. 14-0032-E Mount Sinai Hospital for this study). First trimester, term umbilical cords, and orchiectomy testicular samples were isolated from patients that provided written informed consent. Human granulosa cells (GLCs) and spermatozoa, used as positive controls for the assessment of cell lineage-associated markers, were each obtained from CReATe Fertility Centre patients that provided written informed consent (University of Toronto REB No. 29237 and REB No. 29211, respectively). All animal work was performed in accordance with CACC guidelines and the Toronto Centre for Phenogenomics’ animal facility approval (AUP No. 17-0245 and No. 16-228H, respectively).

### Cell isolation and culture

Previously established male lines of FTM human umbilical cord perivascular cells (*n* = 3) and counterparts derived from full-term birth (*n* = 3; Life Line Stem Cell, New Haven, Indiana, USA) were expanded in α-minimum essential medium (αMEM; Gibco, USA) plus 10% fetal bovine serum (FBS; Hyclone, USA, Lot AWK24007) as described previously (Hong et al., [22]). NTERA2 (pluripotent human testicular embryonal carcinoma cell line clone D1; ATCC, USA, Cat. CREL-1973, Lot 59348173) were used as positive controls in the real-time polymerase chain reaction (RT-PCR) and integrated cell culture (ICC) assessment of GC marker expression including DAZL, VASA, and GPR125. NTERA2 cells were expanded in formulated Dulbecco’s modified Eagle’s medium (DMEM; ATCC, USA). Human testicular tissue was also used as a positive control in some of the immunofluorescence studies (to assess SYCP3 expression). Testicular tissue was cut into 2–4 mm^3^ pieces and the tubules were teased apart with forceps in DMEM/F12 (Gibco, USA) containing nonessential amino acids (ThermoFisher Scientific, USA), 4 mM l-glutamine (ThermoFisher Scientific, USA), sodium bicarbonate (7.5%; Sigma-Aldrich, USA), 40 μg/ml gentamicin (ThermoFisher Scientific, USA), and penicillin (100 IU/ml)/streptomycin 100 μg/ml (ThermoFisher Scientific, USA). Two enzymatic digestion steps were performed by adding 1 mg/ml collagenase type I (Sigma-Aldrich, USA, Cat. LS004196), 1 mg/ml hyaluronidase type II (Sigma-Aldrich, USA, Cat. H2126), and 1 mg/ml trypsin (Sigma-Aldrich, USA, Cat. T1005) for 15 min at 32 °C with gentle shaking followed by 1 mg/ml collagenase type I and 1 mg/ml hyaluronidase for 30 min at 32 °C with gentle shaking. DNase I (8 mg/ml; Sigma-Aldrich, USA, Cat. DN25-7100MG) was used in all steps of isolation. Human granulosa cells (GLCs) were used as positive control for the assessment of FSHR expression, and human spermatozoa were used as positive control for ACROSIN, PRM1, and ODF2 expression, and haploidy.

**Fig. 1:**
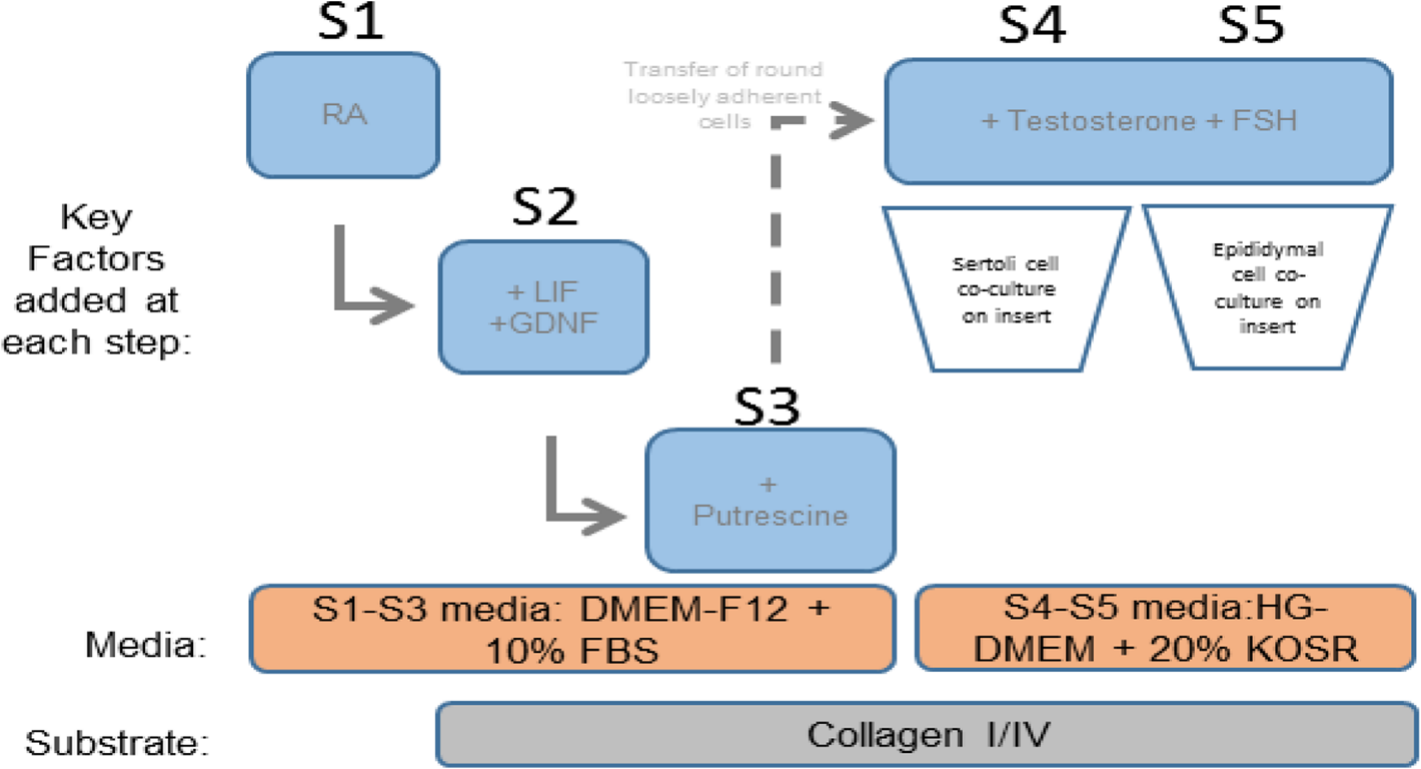
Schematic of the optimized step-wise 5-week differentiation protocol that we developed for HUCPVC differentiation towards the Sertoli and germ cell lineages. *DMEM* Dulbecco’s modified Eagle’s medium, *FBS* fetal bovine serum, *FSH* follicle-stimulating hormone, *GDNF* glial cell-derived neurotrophic factor, *HG* high glucose, *KOSR* knockout serum replacement, *LIF* leukemia inhibitory factor, *RA* retinoic acid

To induce differentiation towards the germ cell and Sertoli cell lineages, FTM and term HUCPVCs (passage 4; *n* = 3 independent lines of each) were first expanded for 2 passages in αMEM + 10% FBS + 5 ng/ml fibroblast growth factor 2 (FGF2; Peprotech, Israel), and then exposed to a 5-step differentiation protocol (Fig. 1) that took place over the course of 5 weeks. This protocol was designed and optimized based on a literature review of testis development and recent in vitro spermatogenesis literature [24–27] to mimic physiologic phases of gonadal development. In Step 1, HUCPVCs were plated at a density of 2.4 × 10^3^ cells/cm^2^ and cultured for 1 week in S1-S3 medium consisting of DMEM-F12 (Gibco, USA) supplemented with 10% FBS, 2 mM l-glutamine (Gibco), 0.1 mM β-mercaptoethanol (Sigma, USA, N0635), 100 mg/ml penicillin/streptomycin, 5 ng/ml epidermal growth factor (EGF; Peprotech, Israel), 5 ng/ml FGF2 (Peprotech, Israel), 10 mM cell culture-tested nicotinamide (Sigma, USA), 2 mM retinoic acid (Sigma, USA), and 1 ml/ml insulin-transferrin-serine solution (ITS; Life Technologies, USA). This medium was changed on day 4 of every week. In Step 2, 4.8 × 10^3^ cells/cm^2^ were plated on culture dishes (BD Biosciences, USA) coated with collagen solution (Stem Cell Technologies, Canada) diluted in phosphate-buffered saline (PBS, 1:40; Sigma, USA) and cultured in S1-S3 media supplemented with 10 ng/ml human leukemia inhibitory factor (LIF; Millipore, USA). In Step 3, 4.8 × 10^3^ cell/cm^2^ were plated on collagen-coated plates and cultured for 1 week in S1-S3 media supplemented with 40 ng/ml glial cell-derived neurotrophic factor (GDNF; Sigma-Aldrich, Canada) and 60 mM putrescine (Sigma, USA). In Step 4, the small round nonadherent cells that developed in Steps 1–3 were isolated and transferred to dishes coated with collagen solution (Stem Cell Technologies, Canada) diluted in PBS (1:40) and co-cultured with adult mouse Sertoli cells placed into 1.0-μm collagen I-coated Transwell™ inserts (VWR, USA) to prevent mixture of human and murine cells. The small round cells were grown in S4-S5 media consisting of knockout DMEM (Gibco, USA) supplemented with 20% knockout serum replacement (Gibco, USA), 1× MEM vitamin solution (Life Technologies, USA), 1× MEM nonessential amino acids (Life Technologies, USA), 2 mM l-glutamine (Gibco, USA), 0.1 mM β-mercaptoethanol (Sigma, USA), 100 mg/ml penicillin/streptomycin (Gibco, USA), 1000 IU/ml follicle-stimulating hormone (FSH; EMD Serono, USA), 40 ng/ml GDNF (Sigma-Aldrich, Canada), 2 mM retinoic acid (Sigma-Aldrich, Canada), 1 mM testosterone (Sigma-Aldrich, Canada), and 0.5 ng/ml human insulin-like growth factor 1 (IGF-1; Sigma-Aldrich, Canada). In Step 5, round cells were co-cultured with primary adult mouse epididymal cells for one week in S4-S5 media. For analysis, mouse cells in the transwell were discarded and only human cells were further analyzed.

### Primary mouse Sertoli cell and epididymal cell cultures

Epididymis and testes were isolated from euthanized adult (4–6 weeks) CD-1 male mice (Charles River). Mouse Sertoli cells and epididymal cells were isolated and cultured as previously described [28].

### Busulfan-induced xenograft model and histological analysis

Six-week-old NOD/SCID mice (*n* = 3; Charles River, USA) were treated 44 mg/kg busulfan (Sigma, USA) at least 6 weeks before donor cell transplantation. FTM HUCPVCs from the end of Step 3 of our protocol were labeled by incubating them with 0.4 μl PKH26 Red Fluorescent (Sigma, USA)/100ul diluent C (Sigma, USA)/1 × 10^6^ cells, as per manufacturer’s instructions. Labeling was qualitatively verified by fluorescence microscopy using the EVOS microscopy (ThermoFisher, USA). For cell transplantation, the seminiferous tubules of recipient mice were filled with approximately 10–15 μl/testis of the donor cell suspension (containing 1 × 10^6^ labeled cells) by injection through the rete testis as described previously [29]. As a control, we injected 10 μl/testis of saline to busulfan-treated mice in the same way. Animals were euthanized by CO_2_ asphyxiation 6 weeks post-cell transplantation and the testes were harvested and snap frozen in OCT (Electron Microscopy Sciences, USA). Unfixed frozen sections were prepared and stained with Hoechst to visualize endogenous PKH26 fluorescence from injected cells and subsequently fixed with 4% paraformaldehyde (PFA) for immunostaining with rabbit anti-human DAZL antibody (1:100; Abcam, USA, Cat. ab34139). The location of the PKH26-labeled cells was analyzed by manual counting of 36 independent tissue sections.

### Gene expression analysis (gene arrays, RT-PCR, qPCR)

Cells were isolated by trypsinization (see above) and cell pellets were snap frozen. RNA samples were prepared using the RNAeasy microkit (Qiagen, USA) according to the manufacturer’s instructions, followed by DNase I treatment to eliminate traces of genomic DNA. For real-time polymerase chain reaction (RT-PCR), RNA was reverse transcribed into cDNA using the RT^2^ First Strand Kit (Qiagen, USA) according to the manufacturer’s instructions. RT-PCR primers are described in Additional file 1: Table S1. A commercial Human Growth Factor RT^2^ Profiler™ PCR Array (PHAS-041ZR-12, Qiagen, USA) was used to evaluate the expression of paracrine factors expressed by undifferentiated FTM and term HUCPVCs cultured in αMEM + 10% FBS. Germ cell lineage-specific genes were assessed using SYBR green quantitative (q)PCR. Briefly, RNA was reverse transcribed into cDNA using RNA to cDNA EcoDry Premix (Clontech, USA, Cat. ST0335, Lot 1310097A) according to the manufacturer’s instructions. All qPCR experiments were performed following the manufacturer’s instructions on the Rotor-gene 6000 instrument (Corbett, USA) using 10 ng total cDNA per reaction. For the qPCR arrays, gene expression levels were determined using PCR Array Data Analysis Centre software (Qiagen, USA) using the delta-delta Ct (ΔΔCt) method and reported as the fold-change relative to term HUCPVCs. For the qPCR-SYBR green primer assays analysis (Additional file 2: Table S2), standard curves were generated using positive control cDNA dilutions [30] and this method was used to report fold-change between undifferentiated and differentiated cells. *GAPDH* and *RPL13* were used as normalizers. All gene array assays were performed in triplicate for at least three independent HUCPVC lines at passage 4. For qPCR of specific germ cell markers, two representative lines of FTM and term HUCPVCs were used. Genes with normalized Ct >30 were considered as not detected.

### Collection and analysis of conditioned media by ELISA

Two hundred thousand HUCPVCs plated on a 10-cm^2^ dish (BD Biosciences, USA) were cultured in αMEM (Gibco, USA) + 10% FBS (Hyclone, USA) until they reached 70% confluency, at which point they were rinsed twice with PBS, and incubated in unsupplemented DMEM-F12 (Gibco, USA) or StemPro 34 (Gibco, USA) medium for 1 h. Unsupplemented basal medium was changed and cells were incubated for 72 h. Medium was collected, filtered using 70-μm cell strainers (Fisherbrand, USA) and snap frozen in 1–5 ml aliquots. Cells were harvested and counted using the Countess® Automated Cell Counter (Life Technologies, USA). For enzyme-linked immunosorbent assay (ELISA), conditioned medium was thawed and concentrated using protein concentrators (Pierce, USA). Basal medium was used as a control. ELISA analysis for human bone morphogenetic protein 4 (BMP4), LIF, basic fibroblast growth factor (bFGF), and GDNF was performed according to manufacturer instructions, including the kit standards (RayBiotech, USA), and analyzed in duplicate on a Multi-Mode Microplate reader F5 (Molecular Devices, USA) The blank optical density (OD) value measured for all experiments was subtracted from basal medium, FTM, and term HUCPVC conditioned medium OD values. The quantity of each factor was calculated using the standard curve equation, dilution factor, and final volume collected, and is expressed as the amount of each factor secreted from the originally plated 200,000 cells.

### Flow cytometry

For analysis of cells throughout the stages of in vitro differentiation, single cell suspensions were obtained by dissociation with TrypLE (Invitrogen, USA) at 37 °C for 5 min and resuspended in 1% FBS/PBS. The cells were filtered through a 70-μm cell strainer (Fisherbrand, USA). Antibodies used include: anti-human FSHR (1:25; Santa Cruz Biotech, USA, Cat. sc-13935), anti-GPR125 (1:80; Cat. ab51705), anti-human GDNFR (1:50; Cat. ab84106) anti-VASA (1:25; Cat. AB13840, all from Abcam, USA). For all reactions, the secondary antibody used was Alexa 488 goat anti-rabbit (1:2000; Life Technologies, USA). Propidium iodide (PI, 1:2000; Sigma, USA) was used to exclude dead cells. Unstained samples were used as controls. Live cells were analyzed for cell surface markers using an LSRII cell analyzer (Becton Dickinson Immunocytometry Systems, USA). Analysis was performed using FlowJo X (Flow Jo, USA). To determine if induced HUCPVCs had undergone meiosis during differentiation, we performed flow cytometric analysis-based DNA content evaluations of FTM and term HUCPVCs throughout the differentiation process [31]. To detect haploid cells, cells were suspended at approximately 10^5^ cells in 0.25 ml PBS (Sigma, USA) and then vortexed to obtain a monodispersed cell suspension. Cell were fixed in 1 ml 70% ice cold ethanol and kept for 1 h on ice. The cells were washed and resuspended in PI solution: 10 μg/ml PI (Sigma, USA) and 100 μg/ml DNase-free RNase A (Sigma, USA, Cat. R-6513) in PBS.

### Immunocytochemistry

Staining with primary antibodies was identical to the above flow cytometry preparation. After primary antibody incubation and washes, cells were placed on Superfrost® Plus slides (MENZEL-GLASER, Germany), allowed to dry for 10 min, and fixed with 4% PFA (Sigma-Aldrich, Canada), followed by secondary antibody staining and mounting with permafluor (ThermoFisher, USA). For DAZL (1:100; Abcam, USA, Cat. ab34139), GPR125 (1:200; Abcam, USA, Cat. ab51705), and FSHR (1:50; Santa Cruz, USA, Cat. sc-13935) immunostaining, cells were washed and fixed in 4% PFA for 15 min prior to the blocking step. For intracellular staining, cells were permeabilized with 0.03% Triton-X (Sigma-Aldrich, USA) in PBS after fixation. Cells were blocked with 10% normal goat/rabbit serum (Jackson Immunoresearch Laboratories, USA) in PBS for 30 min and incubated at 4 °C overnight with the primary antibodies anti-Sall4 (1:250; Abcam, USA, Cat. ab57577), anti-Acrosin (1:50; Santa Cruz, USA, Cat. sc-46284), anti-Protamine1 (1:50; Santa Cruz, USA, Cat. sc-23107), and anti-ODF2 (1:200; Abcam, USA, Cat. ab43840). The cells underwent three 5-min washes with PBS and were incubated at room temperature for 1 h with secondary antibodies Alexa 488 Goat anti-rabbit or rabbit anti-goat (both 1:500; Invitrogen, USA). Nuclei were counterstained with Hoechst (1:1000; Invitrogen, USA) for 10 min followed by three 5-min washes with PBS. Slides were protected with mounting media (PermaFluor, ThermoScientific, USA). Fluorescence images were observed and captured using the EVOS fluorescence microscope (AMG, USA) or spinning disk confocal microscope (Quorum Zeiss AxioVert, SickKids Imaging Facility, Toronto), Images were processed and quantified using Volocity™ (Perkin-Elmer, USA) imaging software.

### Fluorescence in-situ hybridization (FISH)

Cells were collected and then suspended in PBS (Sigma-Aldrich, USA). Carnoy’s Solution (3:1 methanol:acetic acid; Sigma-Aldrich, USA) was added in a 10:1 ratio with the sample suspension, then centrifuged for 5 min at 1500 rpm (300 g). The cell pellet was resuspended in Carnoy’s Solution, incubated for 15 min at –20 °C, and then centrifugation was repeated. Cold 10-μl drops were placed on pre-coated fluoro slides (Thermo Scientific, USA) and warmed to 52 °C. Slides were dehydrated, then decondensed in 0.5 M NaOH (Sigma-Aldrich, USA) solution for 1 min and then dehydration was repeated. Slides were then treated with 4 μl AneuVysion Multicolor DNA Probe (Vysis CEP 18/X/Y; Abbott Molecular, USA), and then incubated in a 37 °C humidified chamber for a minimum of 16 h. Following incubation, slides were rinsed in 0.3% NP 40 in 0.4× SSC warmed to 73 °C for 2 min followed by 0.1% NP 40 in 2× SSC for 10 s. Once dry, slides were counterstained with DAPI (Abbott Molecular, USA) and coverslipped. Slides were analyzed for diploidy by examining chromosomes X, Y, and 18 using DAPI, FITC, TxRed, and Aqua channels on an Olympus BX 61 fluorescence microscope and imaged using HCImage software (Hamamatsu Photonics, Japan). Human sperm was used as a positive control for analysis. Slides were stored between analyses and for the long-term at –20 °C.

### Statistical analyses

All results were generated from at least three independent experiments using 1–3 independent FTM HUCPVC lines and 1–3 term HUCPVC lines. The results are presented as mean ± standard deviation. Statistical significance was determined using the Student’s *t* test and differences were considered significant when *p* < 0.05.

## Results

### HUCPVCs are a heterogeneous cell population expressing both testicular niche growth factors and testis progenitor markers

**Fig. 2:**
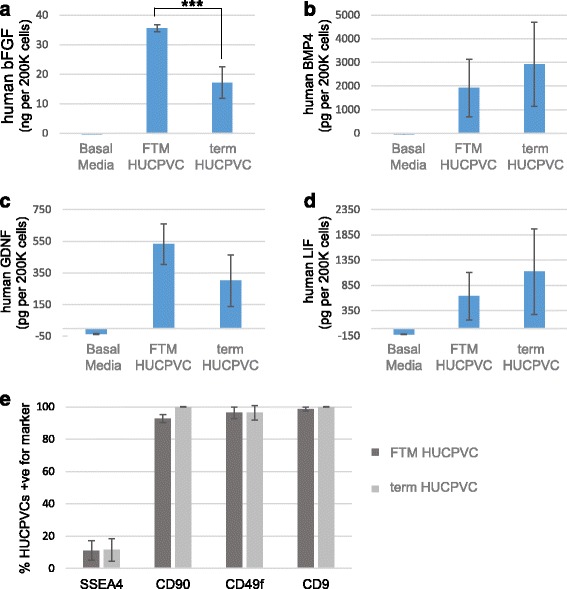
First trimester (*FTM*) and term human umbilical cord perivascular cells (*HUCPVCs*) express and secrete testis niche-associated growth factors. ELISA analysis of the human growth factors **a** basic fibroblast growth factor (*bFGF*), **b** bone morphogenetic protein 4 (*BMP4*), **c** glial cell-derived neurotrophic factor (*GDNF*), and **d** leukemia inhibitory factor (*LIF*) in FTM HUCPVC- and term HUCPVC-conditioned media. Basal media (DMEM-F12) was used as a control. The figure shows the average of two independent lines of FTM HUCPVCs and term HUCPVCs each analyzed in duplicate. Error bars indicate standard deviations. *** *p* = 0.005. **e** Percentage of cells expressing testicular progenitor-associated markers including stage-specific embryonic antigen 4 (*SSEA4*), CD9, CD90, and CD49f as determined by flow cytometry (*n* = 3 independent FTM and term lines each)

FTM and term HUCPVCs expressed similar levels (Ct values between 21 and 27) of genes associated with essential functions in gonadal development and maintenance of the germ cell niche (*p* > 0.05); these included *GDNF*, *LIF*, *FGF1*, *FGF2*, *BMP4* and *BMP6* (Table 1). The levels of GDNF, LIF, FGF2 and BMP4 in FTM and term HUCPVC conditioned medium roughly correlated with the gene expression array data (Fig. 2a–d). Comparable levels of GDNF, LIF, and BMP4 were detected in FTM HUCPVCs and term HUCPVCs, while increased levels of FGF2 were observed in conditioned medium derived from FTM HUCPVCs when compared to that of term HUCPVCs (*p* < 0.05). BMP6 was not detected by ELISA in either HUCPVC type-derived conditioned medium. Interestingly, a subpopulation of undifferentiated HUCPVCs, representing 11.4 ± 7.0%, expressed the SSC-associated marker stage-specific embryonic antigen 4 (SSEA4) [32], and the major population expressed markers previously associated with testicular niche progenitors (CD90, CD49f, and CD9) [33, 34] (Fig. 2e).

**Table 1:** FTM HUCPVCs and term HUCPVCs express testis niche-associated growth factors

Gene	Full gene name	Average Ct		Standard deviation		Fold-change (FTM/term)	*p* value
		Term HUCPVC	FTM HUCPVC	Term HUCPVC	FTM HUCPVC		
GDNF	Glial cell-derived neurotrophic factor	27	27	0.6	0.4	1.08	0.975
FGF2	Fibroblast growth factor 2	22	23	0.2	1.1	0.66	0.693
FGF1	Fibroblast growth factor 1	25.3	26.3	0.32	0.73	0.5	0.037
LIF	Leukemia inhibitory factor	24	24	1.3	0.7	0.95	0.713
BMP6	Bone morphogenetic protein 6	27	27	0.8	1.8	1.39	0.516
BMP4	Bone morphogenetic protein 4	26	28	0.5	2.8	0.28	0.296
TGFB1	Transforming growth factor beta 1	23	23	0.2	0.3	0.83	0.399
VEGFA	Vascular endothelial growth factor A	24	24	0.5	0.9	1.16	0.693

Selected data from the growth factor qPCR array (Sabiosciences) showing transcripts for factors involved in testicular development and function, which are moderately expressed in human umbilical cord perivascular cells (HUPVCs)

Ct values were normalized to the housekeeping genes on the array (*GAPDH, RPL13A, HPRT1*)

*n* = 3 independent lines of first trimester (FTM) HUCPVCs and term HUCPVCs at passage 4 following expansion in α-minimum essential medium + 10% fetal bovine serum

### HUPVCs differentiate into both Sertoli-like and germ-like cells under the same culture conditions in vitro

**Fig. 3:**
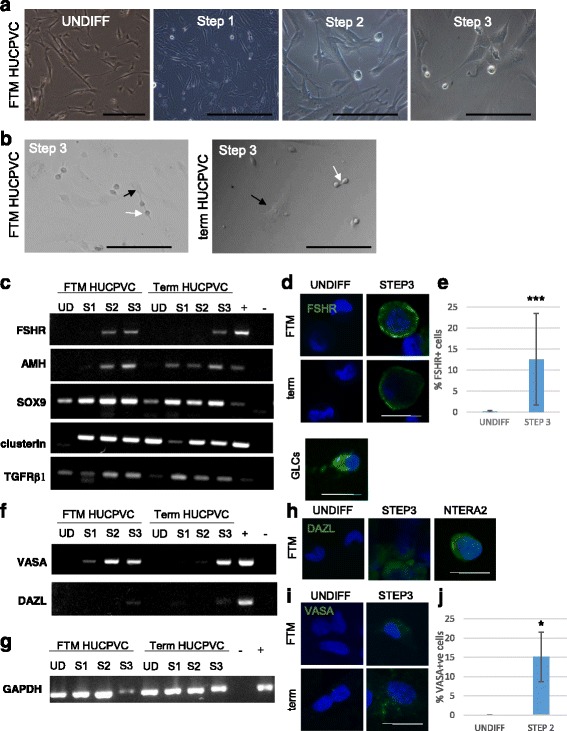
First trimester (*FTM*) and term human umbilical cord perivascular cells (*HUCPVCs*) differentiate towards both Sertoli- and germ-like cells within the same culture environment. **a** Undifferentiated FTM HUCPVCs and FTM HUCPVCs at the end of Step 1, Step 2, and Step 3. **b** FTM HUCPVCs (*left*) and term HUCPVCs (*right*) at Step 3 of the differentiation protocol showing adherent cells (*black arrow*) and small round loosely attached cells (*white arrow*). **c** RT-PCR for Sertoli-associated gene expression including follicle stimulating hormone receptor (*FSHR*), anti-müllerian hormone (*AMH*), *SOX9*, clusterin (*CLU*), and transforming growth factor receptor beta (*TGFR*β*1*) in FTM HUCPVCs and term HUCPVCs at the undifferentiated stage (*UD*), Step 1 (*S1*), Step 2 (*S2*), and Step 3 (*S3*); granulosa cells used as positive control (+). – indicates no cDNA controls. **d** Undifferentiated (*UNDIFF*) FTM HUCPVCs (negative control) (*top, left*) and term HUCPVCs (*bottom, left*) (negative control) immunostained for FSHR (*green*, undetected) and counterstained with Hoechst to show live nuclei. Granulosa cells (*GLCs*) were used as a positive control. **e** Flow cytometric quantification of the percentage of FTM HUCPVCs that upregulated FSHR at the end of Step 2 when compared to undifferentiated controls. **f** RT-PCR for early germ cell-associated gene expression of *VASA* and *DAZL* in the same samples as panel **c. g** RT-PCR for *GAPDH* results shown in panels **c** and **f. h** Undifferentiated FTM HUCPVCs (*left*) immunostained for DAZL (*green*, undetected) and counterstained with Hoechst to show live nuclei. **i** Undifferentiated FTM HUCPVCs (*top, left*) and term HUCPVCs (*bottom, left*) immunostained for VASA (*green*, undetected) and counterstained with Hoechst. **j** Flow cytometric quantification of the percentage of FTM HUCPVCs that upregulated VASA at the end of Step 2 when compared to undifferentiated controls

Based on the postulated mesenchymal origin of Sertoli cells [18], the plasticity of human umbilical cord perivascular cells [22], and their expression of markers of early niche progenitors, we hypothesized that both FTM HUCPVCs and term HUCPVCs could differentiate towards the SC lineage under appropriate in vitro culture conditions. Morphological changes were observed during the first 3 weeks of differentiation (Step 1 to Step 3) when compared to undifferentiated control HUCPVC cultures. Two cell phenotypes were detected: adherent enlarged cells and semi-attached round cells (Fig. 3a and b). Rhodamine dye uptake by the small round cells observed at Step 3 suggests that they are viable (Additional file 3: Figure S1). Gene expression analyses of cells isolated at the undifferentiated stages (negative control), Step 1, Step 2, and Step 3 revealed that, within the same cultures, both SC- and GC-associated genes were upregulated upon differentiation (Fig. 3c, f, and g).

Follicle-stimulating hormone receptor (FSHR) and anti-müllerian hormone (AMH) are specifically expressed in Sertoli cells during testicular development. FSHR expression persists in mature Sertoli cells. We did not detect *FSHR* or *AMH* expression in control undifferentiated FTM HUCPVCs or term HUCPVCs (Fig. 3c and g). In FTM HUCPVCs subjected to this differentiation protocol, *FSHR* was upregulated as early as Step 1 and at each ensuing step, while term HUCPVCs did not upregulate this marker until Step 3 (Fig. 3c and g). Other Sertoli-associated markers (*SOX9*, *TGFR*β*1* and *CLU* (clusterin)) were detected at low levels in undifferentiated HUCPVCs, but were upregulated during the differentiation process (Fig. 3c and g). Upregulation of FSHR expression was observed by immunocytochemistry in HUCPVCs at Step 2 and Step 3 in both FTM and term cells and in granulosa cells utilized as a positive control (Fig. 3d). FSHR was undetectable by flow cytometry in undifferentiated cells (0.17 ± 0. 27%) and was found to be upregulated (5.72 ± 6.32%) among FTM HUCPVCs by the end of Step 2 (*p* < 0.05), and by 12.6 ± 10.8% in FTM HUCPVCs (*p* < 0.001) by the end of Step 3 (Fig. 3e; Additional file 3: Figure S1A). While FSHR was upregulated in some of the experiments using term HUCPVCs (from 0 ± 0% undifferentiated term HUCPVCs to 9.15 ± 9.47% at Step 2 and 3.76% at Step 3), this did not reach significance.

VASA and DAZL are well characterized early germ cell lineage markers [35–37]; *VASA* and *DAZL* were not detected in undifferentiated HUCPVCs from FTM or term HUCPVCs. Unlike FTM cells, *DAZL* gene expression was upregulated as early as Step 1 for term HUCPVC lines, but upregulated in both FTM HUCPVCs and term HUCPVCs by the end of Step 3. In contrast, *VASA* was upregulated at an earlier stage in FTM HUCPVCs (Step 1) when compared to term HUCPVCs (Fig. 3f and g). Upregulation of the GC-associated markers DAZL and VASA was confirmed by ICC (Fig. 3 h and i; lower magnification images shown in Additional file 3: Figure S2) at both Step 2 (data not shown) and Step 3 for both FTM and term cells, as well as in NTERA2 or dissociated human testicular cells (positive controls; Fig. 3h and i; Additional file 3: Figure S3) but not in undifferentiated cells (negative control). Flow cytometric analysis of VASA expression in FTM HUCPVCs in undifferentiated or Step 2 cultures revealed upregulation of this pan-germ cell marker in 15 ± 6.4% of FTM HUCPVCs (*p* < 0.05) (Fig. 3j).

**Fig. 4:**
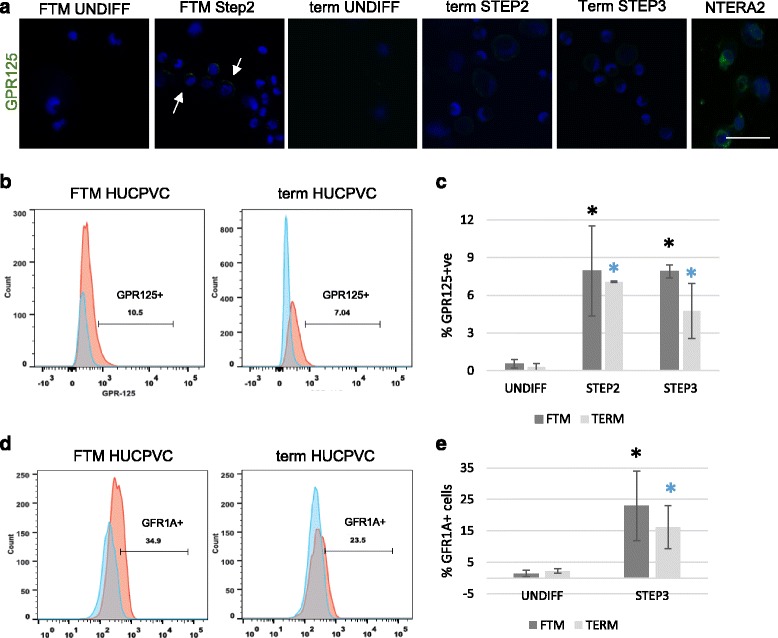
Human spermatogonial stem cell-associated markers are upregulated in first trimester (*FTM*) and term human umbilical cord perivascular cells (*HUCPVCs*) in the early steps of differentiation. **a** Representative micrograph of undifferentiated (*UNDIFF*) FTM HUCPVCs (*left*) and at Step 2 (*second panel*), undifferentiated term HUCPVCs (*third panel*) and at Step 2 and Step 3 (*fourth and fifth panel*, respectively) immunostained for GPR125 (*green*, undetected in undifferentiated PVCs) and counterstained with Hoechst to show live nuclei. GPR125 (*green*) was detected in FTM HUCPVCs and term HUCPVCs at the end of Steps 2 and 3. Human testicular cells were used as a positive control. *Scale bar* = 50 μm. **b** Representative flow cytometry analysis plots for GPR125 in FTM HUCPVCs (*left*) and term HUCPVCs (*right*), comparing the undifferentiated stage to Step 3. **c** Quantification of the percentage of FTM HUCPVCs and term HUCPVCs that upregulated GPR125 at Steps 2 and 3, in comparison to undifferentiated controls (*p* < 0.05 for all comparisons to undifferentiated). *n* = 3 independent lines of each FTM and term HUCPVC. **d** Representative flow cytometry plots for GFR1α expression analysis (also known as GDNFR) in FTM HUCPVCs (*left*) and term HUCPVCs (*right*), comparing the undifferentiated stage to Step 3. **e** Quantification of the percentage of FTM HUCPVCs and term HUCPVCs that upregulated GFR1α at Step 3 in comparison to undifferentiated controls (*p* < 0.05 for all comparisons to undifferentiated). *n* = 3 independent lines of each FTM and term HUCPVC

The spermatogonial stem cell-associated marker GPR125 was significantly upregulated from differentiated FTM HUCPVCs and term HUCPVCs at Step 2 and Step3, when compared to undifferentiated cells used as negative control (*p* < 0.05) (Fig. 4a–c) and human testicular cells used as a positive control (Additional file 3: Figure S3). GDNF receptor (GDNFR), a putative marker of nonhuman primate [38] and human SSCs [39], was also significantly upregulated from undifferentiated to differentiated FTM HUCPVCs and term HUCPVCs at Step 3 (*p* < 0.05) (Fig. 3d and e). There was no significant difference in the upregulation of both of these markers between FTM HUCPVCs and term HUCPVCs (*p* > 0.05).

#### A modified transplantation assay provides evidence for the functional SSC properties of differentiated HUCPVCs

**Fig. 5:**
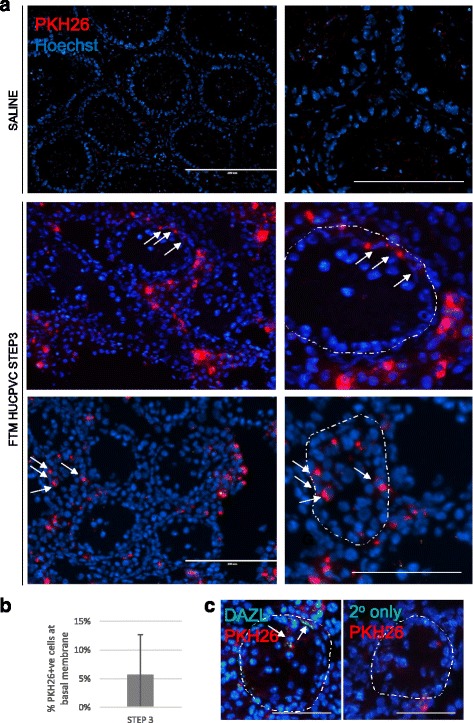
A portion of Step 3-induced first trimester (*FTM*) human umbilical cord perivascular cells (*HUCPVCs*) display functional properties of SSCs in a modified xenograft assay. **a** Representative micrographs of xenograft assay tissue sections including saline control (*top row*, with low magnification on *left* and high magnification on *right*). *Scale bars* = 200 μm and 50 μm, respectively. *Arrows* indicate PKH26-positive cells near the basal membrane of the seminiferous tubule, the contour of which is delineated by the *dotted line*. **b** Quantification of the percentage of total PKH26-positive cells that localize within seminiferous tubules and near the basal membrane as opposed to the interstitial regions. **c** Representative micrograph of DAZL (*green*) immunohistochemistry on frozen sections containing PKH26-labeled human cells (*red*) (*left panel*) and the corresponding no primary antibody negative control (*right panel*). *Scale bar* = 100 μm. *Arrows* indicate PKH26-positive/DAZL+ cells near the basal membrane of the seminiferous tubule, the contour of which is delineated by the *dotted line*

In a busulfan-treated mouse model, functional SSCs injected via the retis testis will migrate to and colonize the basal membrane of the seminiferous tubules [40]. At 6 weeks post-injection, we observed that 5.7 ± 7.1% of fluorescently-labelled Step 3 FTM HUCPVCs localized within tubules near the basal membrane and the remainder of the cells localized to the interstitial spaces (Fig. 5a and b). Some of the intra-tubular cells were also positive for DAZL (Fig. 5c; Additional file 3: Figure S4), suggesting that functional human SSC-like cells populated the mouse tubule.

### Differentiated HUCPVCs undergo spermatid-like morphological changes, together with upregulation of late-stage spermatogenesis markers

**Fig. 6:**
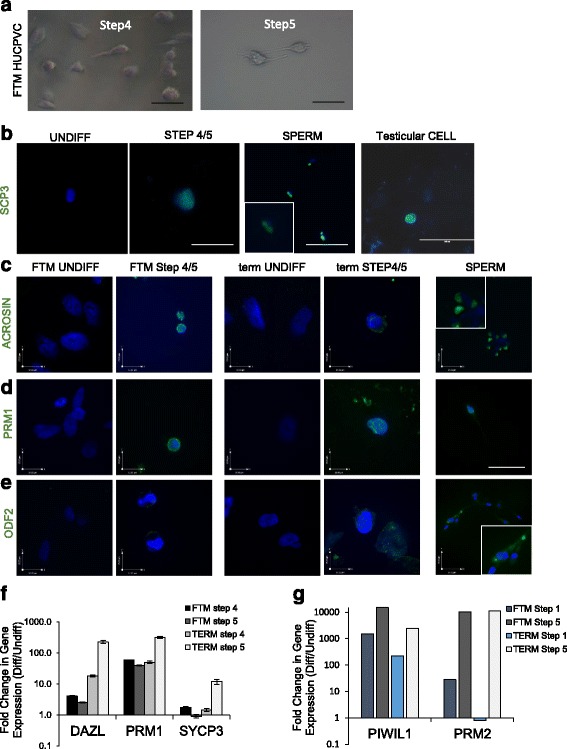
Second stage spermatocyte- and spermatid-associated markers are upregulated in first trimester (*FTM*) human umbilical cord perivascular cells (*HUCPVCs*) and term HUCPVCs at Step 4 and Step 5. **a** Bright field representative micrographs of FTM HUCPVCs at Step 4 and Step 5 of the differentiation protocol. **b** Representative micrograph of undifferentiated (*UNDIFF*) HUCPVCs (negative control) immunostained for SYCP3 (*green*, undetected) and counterstained with Hoechst to show live nuclei. SYCP3 (*green*) was detected in and Step 4 and Step 5 (showing term at Step 5). Spermatozoa (*SPERM*) were used as a positive control. *Scale bars* = 100 μm. **c**–**e** Representative micrographs of undifferentiated (negative control) and Step 5 FTM HUCPVCs and undifferentiated (negative control) and Step 5 term HUCPVCS immunostained for the spermatid-associated proteins Acrosin, Protamine 1 (*PRM1*) and ODF2 (*green*) and counterstained for Hoechst to visualize nuclear integrity. Sperm was used as a positive control. All images are at the same magnification, with scale bars shown, with the exception of insets for sperm markers which were magnified approximately 6 times. **f** qPCR analysis of differentiated (*Diff*) FTM and term HUCPVCs at Step 4 and Step 5 showing fold-change of late spermatogenesis marker (*DAZL*, *PRM1*, *SYCP3*) gene expression when compared to undifferentiated HUCPVCs. **g** qPCR analysis of differentiated HUCPVCs isolated at Step 1 and Step 5, showing fold-change in gene expression compared to undifferentiated cells for early and late spermatogenesis markers *PIWIL1* and *PRM2*, respectively

We observed that, as early as Step 3 and increasingly in Step 4 and Step 5, some of the small round cells developed short tail-like protrusions (Fig. 6a). In addition, the meiosis- and secondary spermatocyte stage-associated marker, SYCP3, was upregulated in both FTM and term HUCPVCs at the gene expression and protein levels in Step 4 and Step 5 (Fig. 6b and f). Spermatid-associated markers including ACR, PRM1, and ODF2 were upregulated in both FTM and term HUCPVCs at Step 4 and Step 5 (Fig. 6c–e) and were not expressed in the undifferentiated HUCPVCs. Interestingly, while both cell types upregulated these markers, the compactness of the nuclei appeared more pronounced in induced FTM HUCPVCs when compared to undifferentiated cells or TERM HUCPVCs (Fig. 6c–e). Gene expression for *DAZL*, *PRM1*, and *SYCP3* were also upregulated in both FTM and term HUCPVCs at Step 4 and Step 5 when compared to undifferentiated cells (*p* < 0.05) (Fig. 6f). *PIWIL1*, an early marker, was markedly upregulated in Step 1 (1000-fold) and in enriched cells at Step 5 in both FTM and term HUCPVC cultures, while the late marker, *PRM2*, was upregulated 10-fold in FTM HUCPVCs at Step 1, but not in term HUCPVCs, when both were compared to undifferentiated cells. *PRM2* was highly upregulated in both cell types by Step 5 (Fig. 6g).

### FTM and term HUCPVCs give rise to haploid cells in vitro

**Fig. 7:**
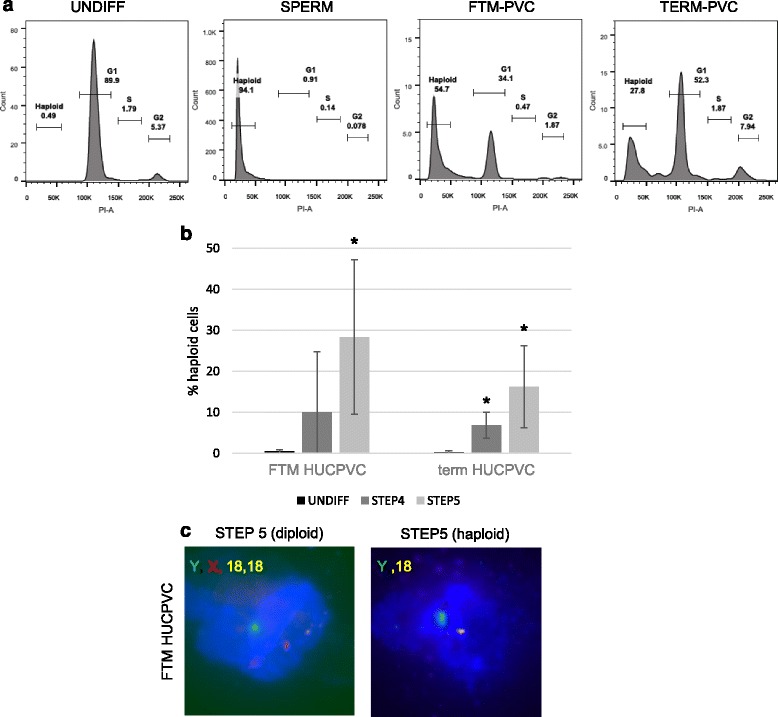
First trimester (*FTM*) and term human umbilical cord perivascular cells (*HUCPVCs*) generate haploid cells. **a** Representative flow cytometry plots for cell cycle analysis of diploid undifferentiated (*UNDIFF*) HUCPVCs (showing FTM HUCPVCs, *left panel*), haploid spermatozoa (*SPERM*, second panel) and FTM HUCPVCs and term HUCPVCs at the end of Step 5 (*right panels*, respectively). **b** Quantification of the percentage of haploid cells in FTM HUCPVCs and term HUCPVCs, comparing undifferentiated HUCPVCs with HUCPVCs at the end of Step 4 and Step 5. Each condition was analyzed in three to four independent experiments. *p < 0.05. **c** Representative micrographs of FTM HUCPVCs processed for fluorescence in-situ hybridization (FISH) at Step 5, showing an example of a diploid cell observed at Step 5 displaying chromosome X (*red*), Y (*green*) and 2 chromosome 18 (*yellow*) and an example of a haploid cells at Step 5 displaying chromosome Y (*green*) and one chromosome 18 (*yellow*)

While undifferentiated FTM HUCPVCs and term HUCPVCs are diploid (99. 5 ± 0.4% vs 99.7 ± 0.3%, respectively; *p* > 0.05), haploid cells were detected at Step 4 and Step 5 in both FTM and term HUCPVCs (28.4 ± 18.8% and 16.2 ± 10.0%, respectively; *p* < 0.05) (Fig. 7a and b). Haploid cells were also detected by chromosome X, Y, 18 FISH in Step 5 FTM HUCPVCs and term HUCPVCs cultures (Fig. 7c).

## Discussion

In this study, we aimed to derive human Sertoli cells from human umbilical cord-derived perivascular mesenchymal stromal cells (HUCPVCs). Unexpectedly both Sertoli-like cells and germ-like cells were derived in vitro under culture conditions designed to mimic those present during normal development. Our findings deliver key advances in conceptual and methodological paradigms for the field of human regenerative reproductive biology research, including possible strategies for the generation of mature functional human spermatozoa.

Our findings that HUCPVCs can differentiate to Sertoli-like and germ cells corroborate previous reports on the ability of MSCs to restore mesenchymal lineages [22, 23] to differentiate to Sertoli cells in mice [41] and their capacity to differentiate into germ cells [42–45]. However, to our knowledge, our results provide the first reported evidence for the differentiation of the human adult stem cell population towards Sertoli-like cells and germ-like cells under the same conditions. Sertoli cells are defined by their phenotypes, including gene and cell surface marker expression, as well as functional properties. Sertoli cells have diverse functions including nursing of germ cells via their secretory products, phagocytosis and elimination of cytoplasm, and as components of a structural blood-testis barrier [46–51]. In the current study, we were able to derive cells which morphologically resembled Sertoli cells, expressed Sertoli-associated genes including *FSHR* and *AMH*, and upregulated *SOX9*, *TGFRB1*, and *CLU*. Furthermore, these cells were able to nurse and support the differentiation of germ cell-like cells in the presence of media supplements.

It remains unclear how both Sertoli-like cells and germ-like cells could be derived under the same conditions from HUCPVCs. Murine studies have demonstrated that germ cells originate from the proximal epiblast [52] while Sertoli cells and HUCPVCs cells originate from the mesoderm and extraembryonic mesoderm, respectively [53]. Germ cell specification requires noncell autonomous signaling by BMPs derived from the extraembryonic ectoderm and visceral endoderm [52, 54–56]. The multipotency of HUCPVCs and their ability to differentiate to two testicular cell lineages under the same culture conditions can likely be attributed to their inherent heterogeneity. Others have identified a subpopulation of very small embryonic-like cells (VSELs) expressing OCT4A and SSEAA [57, 58] from umbilical cord [59, 60] and other tissues [57, 58]. We previously showed that a subpopulation of HUCPVCs express OCT4A and SSEA4 [22], but it remains to be determined whether these cells correspond to previously characterized pluripotent VSELs. In the current study, we further describe at least two distinct subpopulations in our undifferentiated HUCPVC cultures: a minor SSEA4-positive subpopulation and a major SSEA4-negative subpopulation. Each of these cell populations also expressed CD9, CD49f, and CD90, cell surface proteins previously associated with testicular progenitors but also widely reported to be expressed on MSCs [61, 62]. It has been previously reported that SSEA1-positive human umbilical cord-derived MSCs were able to differentiate towards germ cell-like cells [63]. Studies on testis-derived VSELs in a mouse model demonstrated their capacity to differentiate to spermatozoa in vitro [64]. Our results would suggest that the SSEA4-positive subpopulation of HUCPVCs may be equivalent to VSELs, and gave rise to the germ cell lineage differentiation. However, future studies are needed to confirm this hypothesis.

Another important phenotype of undifferentiated HUCPVCs that could explain the duality in their differentiation capacity (to Sertoli-like cells and germ-like cells) is their ability to secrete relevant cytokines and growth factors that promote differentiation to these lineages. In the testicular niche, Sertoli cells play a major role in germ cell differentiation via paracrine secretion of several growth factors. A pivotal growth factor in this process is BMP4 [51] that is secreted by Sertoli cells and activates the SMAD signaling network in germ cells [65]. Previous studies aiming at germ cell differentiation from various sources have supplemented culture media with BMP4 [63, 66, 67], which was not added in our protocol. However, we identified BMP4 as one of the major proteins secreted by undifferentiated HUCPVCs under our growth conditions. Other growth factors that are important in the testicular niche including LIF, GDNF, and FGF2 [12, 49] were also found to be secreted by undifferentiated HUCPVCs; however, supplementation of the differentiation media with these factors appeared to be required for the differentiation of HUCPVCs into germ cell-like cells suggesting that their paracrine properties may be altered under differentiation media conditions (data not shown).

In addition to the heterogeneity and secretory properties of HUCPVCs, we suggest that the growth conditions used in the current study contributed to the differentiation outcome. Similar signaling pathways and growth factors regulate both Sertoli cell and germ cell development [68]. For example retinoic acid (RA), introduced from Step 1 to Step 3, has been demonstrated to induce both the expression of the early germ-specific genes, such as *Stra8*, *Dazl*, and Mvh, and prolonged activation of Smad1/5 in mouse embryonic stem cells [69]. Furthermore, RA promotes Sertoli cell differentiation and antagonizes activin-induced proliferation [70]. RA is also important in the stimulation of germ cells to enter meiosis in the developing mouse ovary. It was also suggested that RA might regulate meiotic progression in the pubertal testis [71].

Our differentiation protocol was designed to mimic physiological spermatogenesis, which is known to take place over 64 days in the human testis. While many parameters of this complex network of events remain unclear, we utilized a novel in vitro model in an attempt to recapitulate the well-defined in vivo stages of spermatogenesis. The small round cells that emerged over the first 3 weeks in culture expressed spermatogonial germ cell-associated markers and were initially diploid (data not shown). To test whether the round cells that were derived after the first three steps of the protocol were spermatogonial stem cells we used the transplantation assay. Engraftment of approximately 5–7% of cells injected in a murine xenograft model at Step 3 suggests that this functional property of SSCs has been induced in our culture system. Although the transplantation assays provide evidence that we were able to derive functional spermatogonia-like cells, under our original conditions these cells could not differentiate any further. The meiotic development of haploid cells occurred under new conditions during weeks 4 and 5. We were able to demonstrate the emergence of gene transcription, protein expression, and morphological changes leading to cells that resembled in vivo human spermatid and spermatocyte differentiation to a degree beyond what has been previously obtained from similar cell sources. As such, this study could contribute to future studies aiming at deriving functional male gametes from HUCPVCs, which we do not claim to have derived here. While we cannot account for cell proliferation in the early steps of differentiation and the loss of differentiated cells at all steps, we estimate that our differentiation efficiency represents approximately 1–3% of the starting cell population.

In the current study, we used two types of HUCPVCs, derived from term and from first trimester umbilical cords. We hypothesized that FTM HUCPVCs, because of their more primitive origins, might possess a broader differentiation capacity as we have previously reported for other lineages. Generally, both cell types demonstrated the same differentiation capacity. However, our qPCR and flow cytometry data suggest that FTM HUCPVCs differentiated faster and more efficiently than term HUCPVCs towards the germ cell lineage. In addition, *DAZL* (a marker of early germ cells) and *SYCP3* (a marker of first meiosis) were both downregulated to a greater degree in FTM HUCPVCs compared to term HUCPVCs at Steps 4 and 5, and this was paralleled by an increased percentage of haploid cell development when compared to term HUCPVCs.

## Conclusion

In conclusion, our study demonstrates the true plasticity and heterogeneity of human umbilical cord perivascular cells which supports their potential for use in male gonadal regenerative therapy applications, including in vitro models of spermatogenesis. Such models would consider the reciprocal relationship between germ cells and their microenvironment (Sertoli cells), thereby mimicking spermatogenesis, and could be used to develop cellular therapies for conditions such as nonobstructive azoospermia. Finally, the paracrine properties of these cells could be harnessed to maintain SSCs or promote their ex vivo/in vivo differentiation in fertility preservation and restoration applications.

## Additional files

Additional file 1: Table S1. RT-PCR primer details. (DOCX 13 kb)
Additional file 2: Table S2. RT^2^ qPCR primer assay details. (DOCX 18 kb)
Additional file 3: Supplementary Figure S1–S4 (PPTX 5065 kb)
