# Supplementary material for: In vitro generation of Sertoli-like and haploid spermatid-like cells from human umbilical cord perivascular cells
Source: Stem Cell Res Ther. 2017 Feb 15;8:37. doi: 10.1186/s13287-017-0491-8 (PMC5312448; doi:10.1186/s13287-017-0491-8)
Supplement: Additional file 1: Table S1. — RT-PCR primer details. (DOCX 13 kb) [file 13287_2017_491_MOESM1_ESM.docx]

| **Gene** | **Sequence** | **Organism** | **PCR Product Size** |
| --- | --- | --- | --- |
| FSHR-F  FSHR-R | TCGTGGGGCTGAGCTTTGAA  GGCTTCCATGAGGGCGACAA | Human | 319 |
| AMH-F  AMH-R | GGCTCTAAGCGCCTATGAG  CTTCCTCCAGGTGTAGGAC | Human | 191bp |
| SOX9-F  SOX9-R | GAGCGAGGAGGACAAGTTC  CATGAAGGCGTTCATGGGC | Human | 151bp |
| Clusterin-F  Clusterin-R | CAGCAGGCCATGGACATC  ATCTCCCGGCACACAGTC | Human | 101bp |
| TGFRβ1-F  TGFRβ1-R | CTGAACCCGTGTTGCTCTC  GCACAACTCCGGTGACATC | Human | 180bp |
| VASA-F  VASA-R | GCCTCTGGGCGGAATTTTGGA  TCGATAGCCGCCTCTCTTGGA | Human | 166bp |
| DAZL-F  DAZL-R | CCTCCTCCACCACAGTTTC  CAGAGGGTGGAGTAGCTTC | Human | 319bp |

**Table S1: RT-PCR primer details**
