# Supplementary material for: In vitro generation of Sertoli-like and haploid spermatid-like cells from human umbilical cord perivascular cells
Source: Stem Cell Res Ther. 2017 Feb 15;8:37. doi: 10.1186/s13287-017-0491-8 (PMC5312448; doi:10.1186/s13287-017-0491-8)
Supplement: Additional file 2: Table S2. — RT2 qPCR primer assay details. (DOCX 18 kb) [file 13287_2017_491_MOESM2_ESM.docx]

| **Gene** | **Catalog #** | **Organism** | **Manufacturer** |
| --- | --- | --- | --- |
| PRM1 | PPH07169A | Human | [Qiagen](https://my.labguru.com/catalog/companies/251) |
| PRM2 | \| PPH07137A \| \| --- \| \|  \| | Human | [Qiagen](https://my.labguru.com/catalog/companies/251) |
| SYCP3 | PPH21252A | Human | [Qiagen](https://my.labguru.com/catalog/companies/251) |
| DAZL | PPH09876B | Human | [Qiagen](https://my.labguru.com/catalog/companies/251) |
| PIWIL1 | PPH19177A | Human | [Qiagen](https://my.labguru.com/catalog/companies/251) |

**Table S2: RT² qPCR Primer Assay details**
