# Supplementary material for: In vitro generation of Sertoli-like and haploid spermatid-like cells from human umbilical cord perivascular cells
Source: Stem Cell Res Ther. 2017 Feb 15;8:37. doi: 10.1186/s13287-017-0491-8 (PMC5312448; doi:10.1186/s13287-017-0491-8)
Supplement: Additional file 3: — Supplementary Figure S1–S4 (PPTX 5065 kb) [file 13287_2017_491_MOESM3_ESM.pptx]

## Slide 1
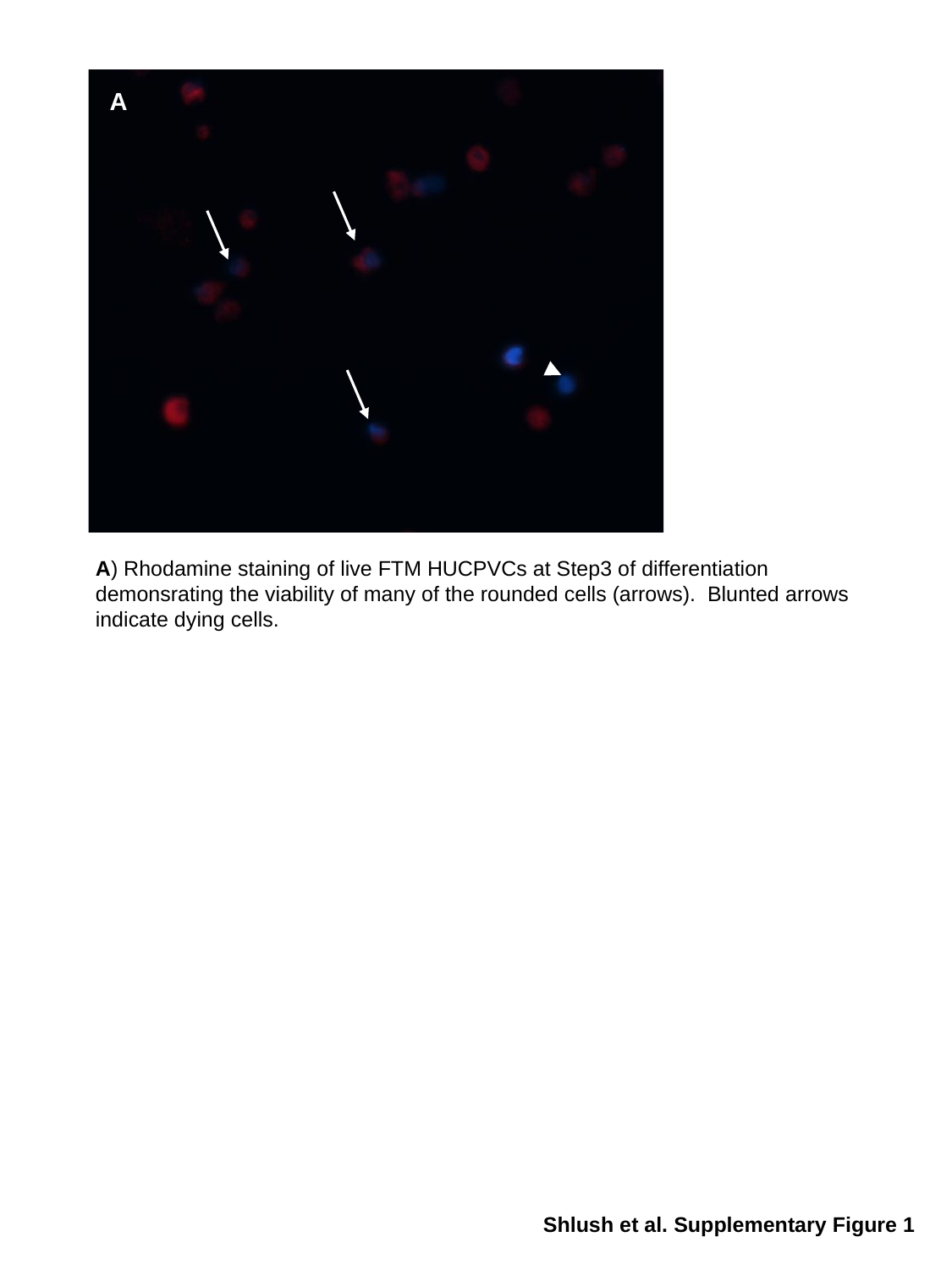

A
A) Rhodamine staining of live FTM HUCPVCs at Step3 of differentiation demonsrating the viability of many of the rounded cells (arrows). Blunted arrows indicate dying cells.
E
Shlush et al. Supplementary Figure 1

## Slide 2
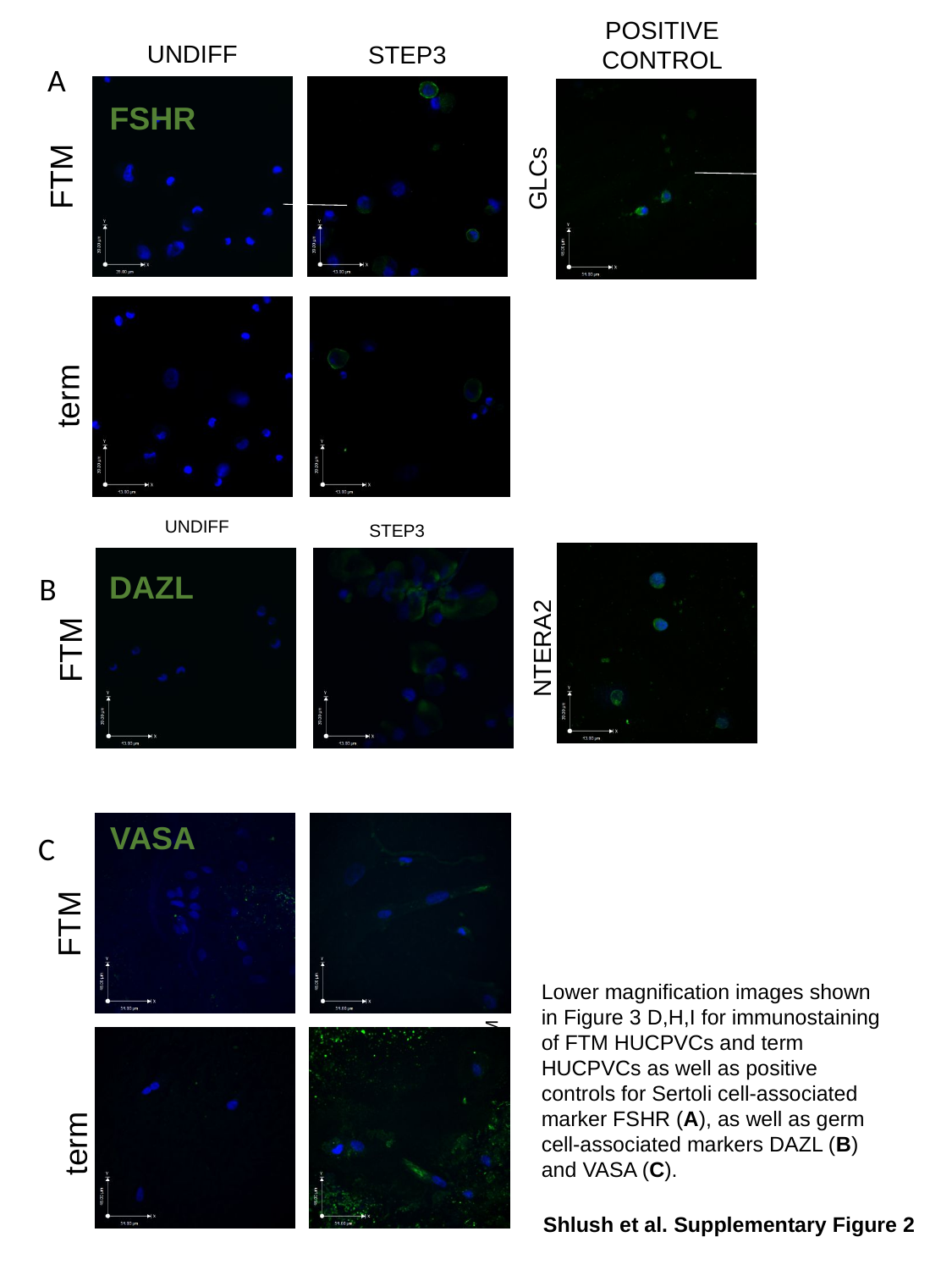

POSITIVE CONTROL
UNDIFF
STEP3
A
FSHR
FTM
GLCs
term
UNDIFF
STEP3
DAZL
B
FTM
NTERA2
VASA
C
FTM
I
Lower magnification images shown in Figure 3 D,H,I for immunostaining of FTM HUCPVCs and term HUCPVCs as well as positive controls for Sertoli cell-associated marker FSHR (A), as well as germ cell-associated markers DAZL (B) and VASA (C).
FTM
term
term
Shlush et al. Supplementary Figure 2

## Slide 3
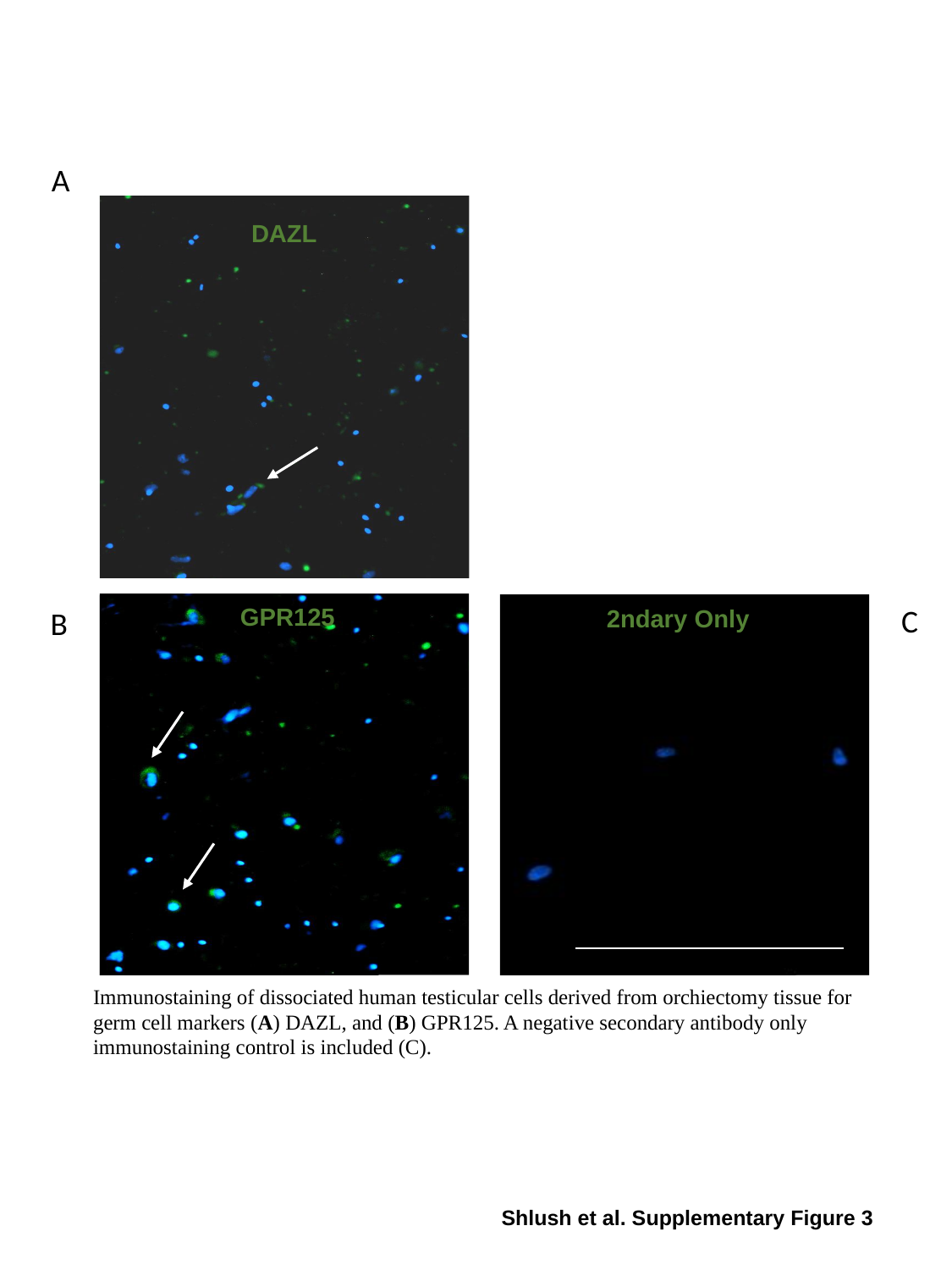

A
DAZL
GPR125
C
B
2ndary Only
Immunostaining of dissociated human testicular cells derived from orchiectomy tissue for germ cell markers (A) DAZL, and (B) GPR125. A negative secondary antibody only immunostaining control is included (C).
Shlush et al. Supplementary Figure 3

## Slide 4
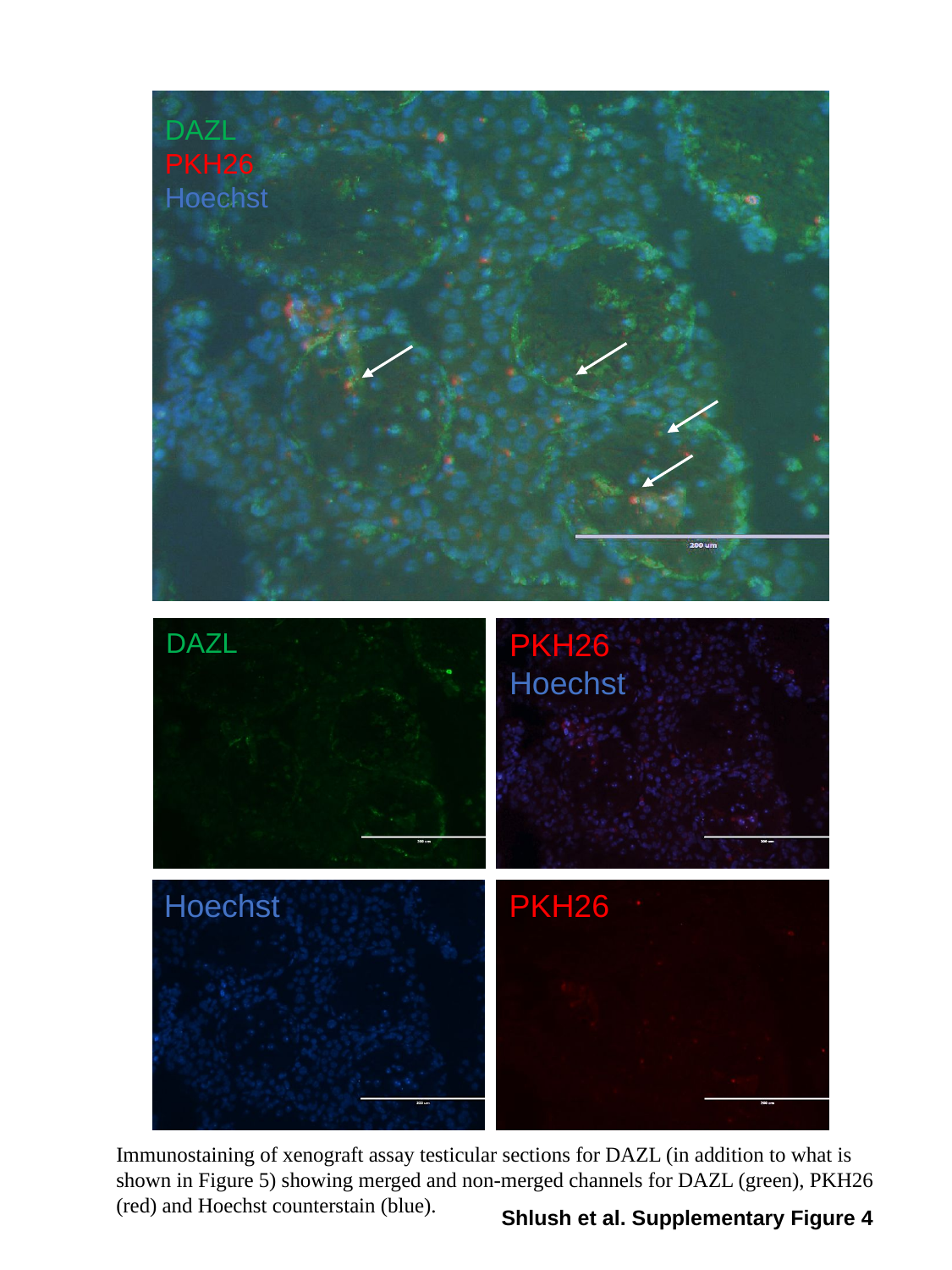

DAZL
PKH26
Hoechst
PKH26
Hoechst
DAZL
Hoechst
PKH26
Immunostaining of xenograft assay testicular sections for DAZL (in addition to what is shown in Figure 5) showing merged and non-merged channels for DAZL (green), PKH26 (red) and Hoechst counterstain (blue).
Shlush et al. Supplementary Figure 4
